# Auricular acupoint therapy for functional gastrointestinal disorders: a systematic review and meta-analysis of randomized clinical trials

**DOI:** 10.3389/fmed.2025.1513272

**Published:** 2025-03-19

**Authors:** Meng-Yuan Shen, Ze-Jiong Li, Shu-Han Wang, Tian-Chen Lin, Qin-Yi Lou, Shan Liu, Dan-Dan Feng, Dong-Dong Yang, Chen-Juan Wang, Zhe-Kai Ying, Rong Zhou, Jian-Nong Wu

**Affiliations:** ^1^The First Affiliated Hospital of Zhejiang Chinese Medical University (Zhejiang Provincial Hospital of Chinese Medicine), Zhejiang, China; ^2^Department of Intensive Care Unit, The First Affiliated Hospital of Zhejiang Chinese Medical University (Zhejiang Provincial Hospital of Chinese Medicine), Hangzhou, China

**Keywords:** auricular acupoint therapy, functional gastrointestinal disorders, systematic review, meta-analysis, randomized controlled trials

## Abstract

**Introduction:**

This study aims to conduct a systematic review and meta-analysis of randomized controlled trials to evaluate the efficacy and safety of auricular acupoint therapy (AAT) for functional gastrointestinal disorders (FGIDs).

**Methods:**

We conducted a thorough search across eight databases, including PubMed, EMBASE, Web of Science, the Cochrane Library, CNKI, Wanfang, VIP, and CBM. The search covered the period from the inception of each database up to June 30, 2024. The authors independently reviewed all the references, evaluated the risk of bias, and extracted the data. GRADEpro software was utilized to calculate overall strength of evidence. A random effects or fixed effects model was selected on the basis of the *p*-value and *I^2^*. RevMan 5.3, Stata/MP 18.0, R 4.3.1 and R Studio 2023.09.0 were used for data processing. TSA 0.9.5.10 beta software was used to evaluate data stability.

**Results:**

The review included 19 randomized controlled trials with a total of 1,681 patients (895 in the treatment group and 886 in the control group). The treatment duration ranged from 2–12 weeks. The meta-analysis revealed that, compared with the control, AAT was significantly more effective at treating FGIDs (RR: 1.35; 95% CI: 1.21–1.51; *p* < 0.001), reducing the symptom score (MD: −1.94; 95% CI: −3.06 to −0.85; *p* < 0.001; five trials), improving the SAS score (MD: −12.47; 95% CI: −13.92 to −11.01; *p* < 0.001; five trials), and improving the SDS score (MD: −4.97; 95% CI: −9.23 to −0.72; *p* = 0.02; six trials). A total of two articles mentioned relatively significant adverse reactions (MD: 2.98; 95% CI: 0.51–17.26; *p* = 0.009). Sensitivity and trial sequential analyses confirmed the stability of these results.

**Discussion:**

While our meta-analysis suggests that AAT may offer benefits for FGIDs, these results must be interpreted with caution due to methodological limitations in the included trials. Further investigations in high-quality trials are warranted.

**Systematic review registration:**

https://clinicaltrials.gov/, identifier CRD42024558786.

## 1 Introduction

Functional gastrointestinal disorders (FGIDs), including functional dyspepsia (FD), functional constipation (FC), and irritable bowel syndrome (IBS). It is characterized by chronic gastrointestinal symptoms without any identifiable organic pathology (1–3). According to the Rome IV criteria, FGIDs are classified as gut-brain interaction disorders. They are divided into eight categories based on the basis of different gastrointestinal symptoms, encompassing a total of 32 distinct conditions (4, 5). FD, IBS, and FC are present worldwide, and their incidence is increasing worldwide. Studies show that FD affects approximately 16% of the global population, significantly impacting patients’ quality of life (6). The prevalence of IBS ranges from 5 to 10%, and that of FC is around 15.3%. These disorders significantly affect individuals’ quality of life, work productivity, and society through frequent medical consultations, medication use, and over-the-counter treatments (7, 8). Thus, effective prevention and treatment strategies for FGIDs are crucial in clinical practice.

The pathophysiological mechanism of FGIDs is complex, and its development is the result of the interaction of physiological, psychological and social factors. At present, the pathogenesis of FGIDs has not been fully elucidated (9, 10). Currently, FGID management relies primarily on gastrointestinal motility agents, along with antianxiety and antidepression treatments. However, its clinical use is limited by long-term side effects, unclear efficacy, and safety concerns associated with various treatments. However, no approach fully addresses the complex pathological changes associated with FGIDs.

Auricular acupoint therapy (AAT), also known as auriculotherapy or auricular therapy, is a key component of traditional Chinese medicine (TCM). AAT has been used in TCM for thousands of years to treat a variety of conditions. It is a non-invasive and drug-free treatment that has gained increasing attention in recent years for its potential to improve symptoms and quality of life in patients with FGIDs. According to TCM theory, specific auricular points (e.g., “large intestine,” “spleen,” and “liver”) corresponding to internal organs and physiological functions. Stimulating these points is thought to regulate qi and blood flow, harmonize Zang-Fu organ systems, and restore gastrointestinal homeostasis (11–13). Modern studies further suggest that AAT can enhance gastric hypersensitivity in rats with FD by balancing sympathetic and vagal nerve activity, suggesting its potential for alleviating gastric pain in FD patients (14). Additionally, stimulating ear points can activate the brainstem to release dopamine, reduce food intake, promote stomach relaxation, and regulate mood (15, 16). These studies show that AAT modulates the brain-gut axis via vagal nerve activation, reducing visceral hypersensitivity and normalizing gut motility—key mechanisms disrupted in FGIDs (17, 18). Additionally, the non-invasive nature and minimal adverse effects of AAT make it an appealing alternative to pharmacological therapies, particularly for chronic conditions requiring long-term management (12). Recent RCTs have demonstrated its efficacy in alleviating FGID symptoms, supporting its integration into evidence-based practice (19, 20).

Considering the current lack of high-quality meta-analyses and the specific impact of AAT on FGID, it is still not fully understood. The purpose of this article is to analyze and summarize the efficacy and safety of AAT in the treatment of FGID thoroughly.

## 2 Methods

This meta-analysis was conducted in accordance with the Preferred Reporting Items for Systematic Reviews and Meta-analysis Statement. The review protocol was prospectively registered in the International Prospective Registration of Systematic Reviews and publicly available (CRD42024558786) and followed without deviation. All planned outcomes, analyses, and subgroup assessments were executed as specified in the registration.

### 2.1 Data sources and search strategies

We conducted a thorough search across eight databases, including PubMed, EMBASE, Web of Science, Cochrane Library, CNKI, WanFang, VIP, and CBM. The search covered the period from the inception of each database up to June 30, 2024. Two investigators (Shu-Han Wang, Ze-Jiong Li) independently screened all records identified through the search and reviewed the full text for eligibility. For studies that met the inclusion criteria, data were extracted independently by two authors and then reviewed by a third author (Qin-Yi Lou). Any disputes will be resolved by the corresponding author (Jian-Nong Wu). This protocol was applied to all database searches, with some modifications to search terms and operators. The search criteria were based on participants, intervention, comparison, outcome, time, and study design (PICOTS), and the search strategy was structured around the search terms “functional dyspepsia, “Irritable Bowel Syndrome, and “functional constipation, and “functional gastrointestinal disorders, and “Auricular acupoint therapy, and “randomized controlled trials, Subject terms, their synonymous free words, and qualifiers were used to improve search sensitivity: (“Indigestion” OR “postprandial distress syndrome” OR “epigastric pain syndrome” OR “indigestion” OR “functional dyspepsia” OR “functional dyspepsia” OR “irritable bowel syndromes” OR “mucous colitis” OR “mucous colitides” OR “syndrome, irritable bowel” OR “syndromes, irritable bowel” OR “irritable colon” OR “colitis, mucous” OR “colitides, mucous” OR “mucouscolitides” OR “functional constipation” OR “dyschezia” OR “colonic inertia” OR “chronic functional constipation” OR “slow transit constipation” OR “chronic severe functional constipation” OR “functional gastrointestinal disorders” OR “functional bowel disease” OR “functional gastrointestinal disease”) AND (“Acupuncture” OR “electroacupuncture” OR “bloodletting” OR “laser” OR “acupressure” OR acupoint” OR “therapy” OR “stimulate” OR press stick needle” OR “auriculotherapy” OR “otopoint” OR “ototherapy OR “Auricular” OR “ear” OR “acupuncture treatments”) AND (“randomized controlled trial” OR “randomized”). In PubMed, search results were limited to “randomized controlled trials.” The search strategy is detailed in Supplementary Table S1.

### 2.2 Study selection and eligibility criteria

The eligibility of studies was determined based on the following criteria: (i) Study Type: RCTs reported in English or Chinese. (ii) Participants: Patients over 18 years old with FGID, diagnosed according to criteria including but not limited to the latest version of the Rome criteria. (iii) Intervention: All forms of AAT that are not combined with other interventions, such as acupuncture, electroacupuncture. (iv) Comparison: The control group includes the regular care group, placebo group, and western medicine group. The placebo group involves the use of non-irritating objects in placebo patches or sham interventions. (v) Outcomes: studies evaluating treatment effectiveness were considered eligible.

Exclusion criteria included the following: (i) Duplication, defined as the same data from subjects in different studies by the same authors; (ii) Unavailability of full text or critical data of the articles was not obtained.

### 2.3 Data extraction and study quality

Two authors independently screened all records identified through the search. They then reviewed the full text for eligibility. Data were extracted by two authors and reviewed by a third author. Any discrepancies were resolved by the corresponding author. The recorded study characteristics included author, country, and year of publication; participant characteristics such as diagnostic criteria, age, gender, and number of cases in each group; intervention information including measures of intervention and control, treatment duration, and adverse events; as well as outcome details such as outcomes, adverse events, and follow-ups. Any differences were settled through discussion. Authors of studies with incomplete or ambiguous reported data were contacted.

The quality of RCTs was assessed using the Cochrane Risk-of-Bias Tool (21), which considered randomization methods, allocation concealment, blinding procedures, data integrity, selective result reporting, and other potential sources of bias. These were categorized as “unknown risk,” “low risk,” or “high risk” based on migration criteria.

The GRADE system (22) was used to rate the quality of evidence for the outcomes assessed. For each outcome, we automatically assigned four points to each study because they were RCTs, and downgraded them if there was an increased risk of bias, inconsistency, indirectness, imprecision, and publication bias. We classified the quality of the evidence as A (high), B (moderate), C (low), or D (very low). Two researchers (Tian-Chen Lin and Ze-Jiong Li) independently assessed the quality of the evidence and resolved any disputes by discussion with a third researcher (Jian-Nong Wu).

### 2.4 Data synthesis and analysis

RevMan 5.3 (Cochrane Collaboration), Stata/MP 18.0 (StataCorp LLC), and R version 4.3.1 (R Foundation for Statistical Computing) with R Studio 2023.09.0 (Posit Software) were used for data analysis. The risk ratio (RR) was used for binary data, while the weighted mean difference (WMD) or standardized mean difference (SMD) was used for continuous data, both with 95% confidence intervals (CI). RR values and their 95% CIs were combined, and routine meta-analyses were performed. Heterogeneity was assessed using the Cochran Q test and *I^2^* statistic (23). If *p* > 0.1 and *I^2^* ≤ 50%, the fixed-effect model was used; if *p* < 0.1 or *I^2^* > 50%, the random-effects model was applied along with subgroup and sensitivity analyses to identify sources of heterogeneity (24, 25). Additionally, a cumulative meta-analysis using the “metacum” package in R Studio examined changes in pooled estimates over time to identify trends and assess accuracy while exploring potential sources of heterogeneity. Publication bias was assessed using funnel plots and confirmed through Egger’s test in Stata/MP 18.0. A significance level of *p* < 0.05 was considered statistically significant (26).

The TSA 0.9.5.10 beta software was used for sequential analysis in the treatment of FGIDs (27). In this study, the Type I error probability was set at *α* = 0.05, and statistical power was 80%. The relative risk reduction was defined as 20% compared to the control group event rate. If the cumulative Z value reached both the traditional threshold (*Z* = 1.96) and the TSA threshold, the results were considered conclusive evidence after correction.

## 3 Results

**Figure 1:**
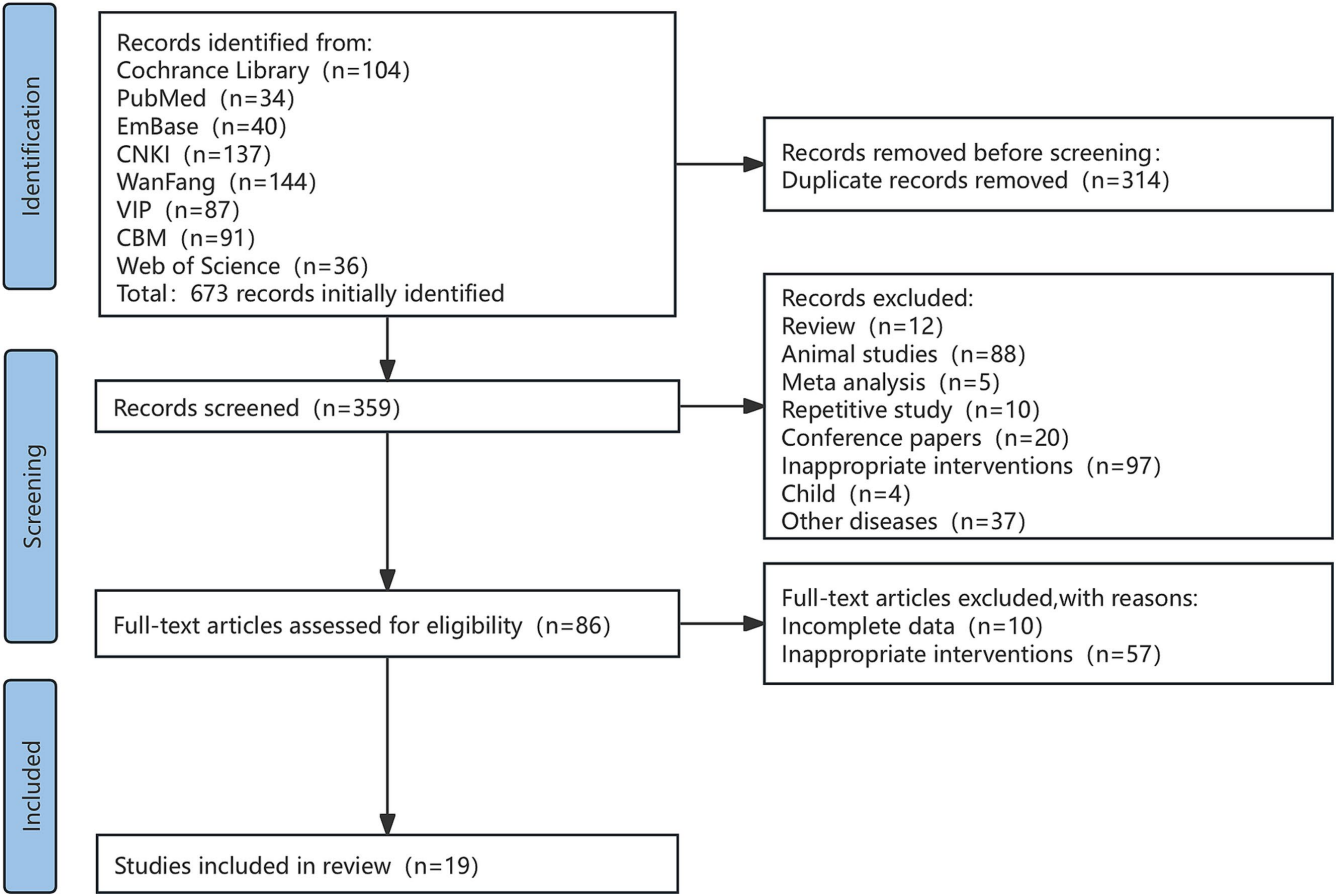
A flowchart of literature search and selection process.

The database search identified a total of 673 studies. After the literature screening process, 654 studies were included for further review. Ultimately, 19 RCTs were selected for our analysis (Figure 1).

### 3.1 Study characteristics and quality assessment

**Figure 2:**
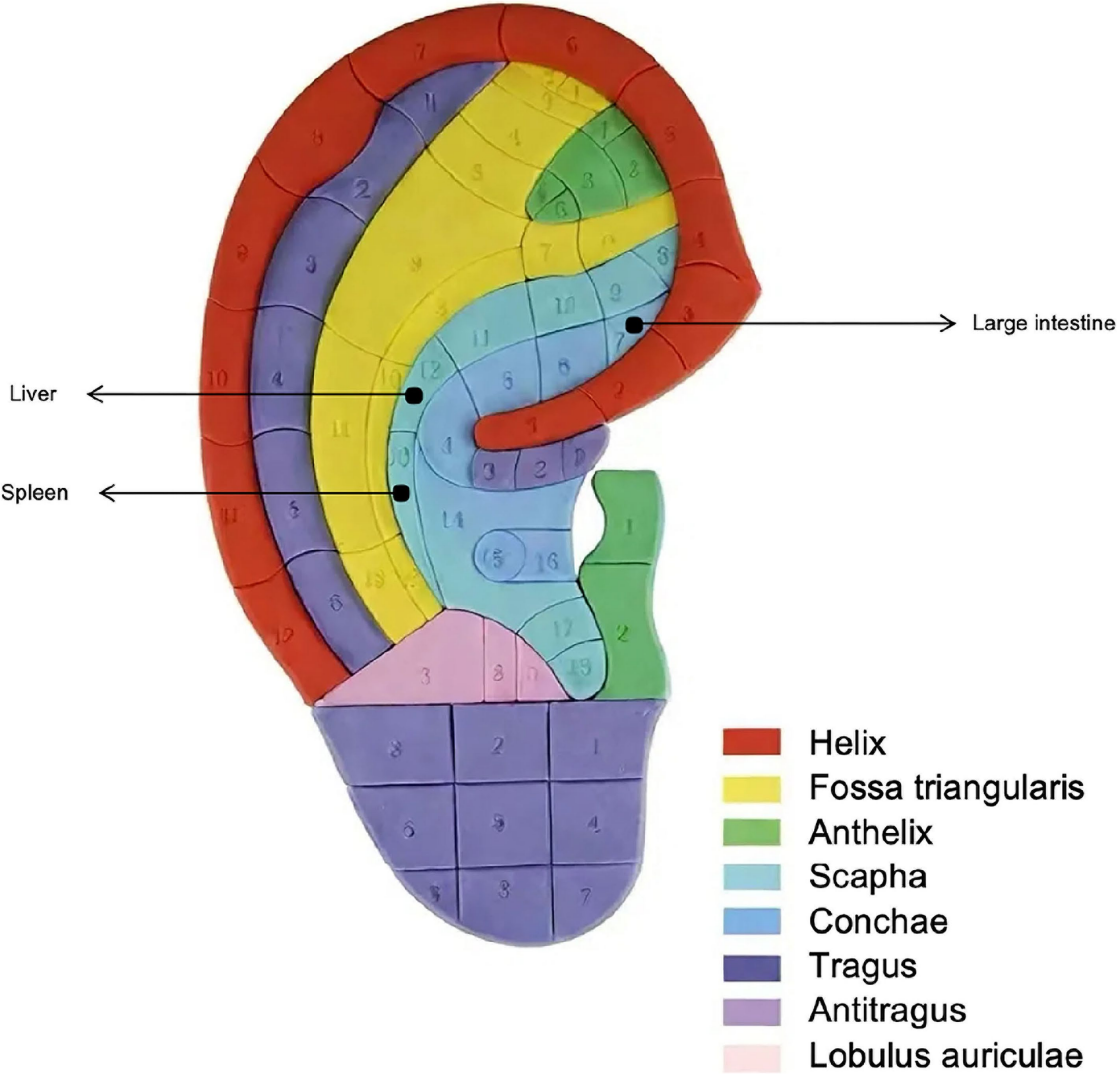
Major acupoints selected to treat FGIDs.

This systematic review included 1,681 individuals diagnosed with FGIDs from 19 RCTs. The participants comprised 895 individuals in the treatment group and 886 in the control group. All included studies, including English articles, were conducted in China. The studies were first published between 2006 and 2023 and the sample size varied between 20 and 300. The mean age of participants ranged from 21.6 to 75.8 years, and there was no significant bias in the gender distribution. The diagnostic criteria for 2 studies were Rome III (28, 29), 5 studies were Rome III (30–34) and 7 studies were Rome IV (35–40). Some studies use non-Rome criteria (41, 42) or are not explicitly stated (43–45). Six studies focused on FD (30, 35–37, 41), five focused on IBS (31, 38, 39, 42, 43), and eight focused on FC (28, 29, 32–34, 40, 44, 45). Auricular acupuncture points were applied unilaterally in one trial, bilaterally in 15 trials, and were not specified in three trials. The most commonly used acupuncture points include the large intestine (11 items), spleen (8 items), and liver (7 items). The interventions primarily involved AAT in the form of acupressure (13 trials) or electroacupuncture (6 trials). The intervention time and frequency of AAT varied according to the specific type, ranging from 2 weeks to 12 weeks, and the frequency of intervention from 3 times weekly (35, 38) to 5 times daily (29–31, 33, 40). 2 RCT (36) did not explicitly explain the frequency of acupuncture therapy interventions. Six studies used usual care, six studies used placebo, including the sham group, and seven studies used conventional Western medicine (include Moxapride, domperidone, etc.). An overview of the characteristics of all included studies and meta-analysis is provided in Table 1. The locations of the acupoints are detailed in Figure 2.

**Table 1:** Characteristics of the included trials.

References	Sample size (E/C)	Age: mean ± SD OR min-max (mean) (E/C)	Sex(M/F)	Diagnostic criteria	Disease duration (E/C)	Intervention duration (week)	Intervention method	Frequency of AAT sessions	Acupoint	Control method	Outcome measures	Adverse event (patients, n)
Lin et al. (41)	35/35	21–60(38.7)/20–65(39.5)	E: 16/19; 15/20	Non-Rome	E: 2 M-10Y; C: 3 M-9Y	4	Acupressure	3 times daily post-meals, 1 min/each application	Shenmen(TF4); Pi(CO13); Wei(CO4); Gan(CO12)	Domperidone	Efficacy	1
Wang et al. (30)	30/30	NA	NA	Rome III	NA	4	Acupressure	at least 5 times daily, 1 min/each application; 3 times weekly, with at least a 1-day interval between each application.	Pi(CO13); Wei(CO4); Gan(CO12); Shen(CO10); Shierzhichang(CO5); Neifenmi(CO18); Jiaogan(AH6a); Shenmen(TF4); Pizhixia(AT4)	Mosapride Citrate Dispersible	Efficacy; NDSI; NDLQI	NA
Wu et al. (35)	45/45	51.56 ± 6.88/52.04 ± 7.37	E: 18/27; 16/29	Rome IV	E: 6.18 ± 3.69Y; C: 7.51 ± 3.35Y	4	Electroacupuncture	3 times weekly	Left ear cavum conchae	Sham-taVNS	Efficacy; Overall symptom score; FDDQL; HAMA; HAMD; SDS	NA
Shi et al. (36)	200/100	NA	NA	Rome IV	NA	4	Intervention A:10 Hz electroacupuncture; Intervention B: 25 Hz electroacupuncture	NA	left ear screens	Sham-taVNS	Efficacy	7
Zhou et al. (37)	26/28	28.0–51.75/27.5–49.75	E: 6/20; 7/21	Rome IV	E: 6-120 M; C: 12-114 M	2	Electroacupuncture	2 times daily	Left tympanic cavity	Sham- taVNS	Efficacy; Overall symptom score; NDSI; HAMA; SAS; SDS	NA
Wu et al. (36)	45/45	50.58 ± 8.75/48.31 ± 9.31	E: 16/29; 12/33	Rome IV	E: 4.82 ± 2.98Y; C: 5.23 ± 2.86Y	12	Electroacupuncture	NA	Left ear cavum conchae	tnVNS	Efficacy; Overall symptom score; FDDQL; HAMA; HAMD; SDS	No
Hang et al. (43)	40/40	42.05 ± 8.8741.72 ± 8.03	E: 18/22; 16/24	NA	E: 10.31 ± 1.89 M; C: 10.56 ± 2.01 M	4	Acupressure	4 times daily	Wei(CO4); Dachang(CO7); Gan(CO12); Pi(CO13); Neifenmi(CO18); Pizhixia(AT4)	Montmorillonite Powder	Efficacy; TCM syndrome scale; SAS; SDS	No
Huang et al. (31)	32/32	20–60/19–61	E: 18/14; 19/13	Rome III	E: 1.2–6.5Y; C: 1.3–6.6Y	4	Acupressure	3–5 times daily, 6 days a week	Zhichang(HX2); Dachang(CO7); Jiaogan(AH6a); (Shenmen) TF4; Neifenmi(CO18); Pizhixia(AT4); Gan(CO12); Pi(CO13);	Pinaverium Bromide	Efficacy; Serum 5-HT	No
Kang et al. (42)	54/46	NA	E: 32/22; 27/19	Non-Rome	6 M- 15Y	4	Acupressure	3–4 times daily	Xin(CO15); Gan(CO12); Pi(CO13); Wei(CO4); Shenmen(CO10); Dachang(CO7); Xiaochang(CO6)	Dicycloverine	Efficacy	No
Wu et al. (38)	41/41	38.27 ± 9.22/41.97 ± 8.77	E: 11/30; 10/31	Rome IV	E: 4.82 ± 2.98Y; C: 5.23 ± 2.86Y	12	Electroacupuncture	3 times weekly	Gan(CO12); Pi(CO13)	Sham-taVNS	Efficacy; IBS-SRS; IBS-SSS; HAMA; HAMD; SF-36	No
Shi et al. (39)	21/19	41.5 ± 15.4/49.6 ± 15.6	NA	Rome IV	NA	4	Electroacupuncture	NA	Left ear cavum conchae	Sham-taVNS	Efficacy; CSBM; VAS; SDS; SAS; BSFS; IBS-QOL; IBS-SSS	No
Huang et al. (44)	75/75	NA	NA	NA	NA	4	Acupressure	2–3 times daily	Pizhixia(AT4); Sanjiao(CO17); Dachang(CO7); Zhichang(HX2); Gan(CO12); Pi(CO13); Shen(CO10); Yidan(CO11); Wei(CO4)	Lactulose	Efficacy; BSFS; CSBM; PAC-SYM; PAC-QOL	No
Ji et al. (28)	37/36	72.30 ± 1.07/69.81 ± 1.04	E: 19/18; 18/18	Rome II	NA	4	Acupressure	3 times daily	1.Mainpoint:Dachang (CO7); Xiaochang(CO6); Zhichang(HX2): 2.Actual symptoms: Fei(CO14); Sanjiao(CO17); Wei(CO4). 3.Deficiency symptoms: Pi(CO13); Shen(CO10); Neifenmi(CO18)	Routine treatment	Efficacy; PAC-SYM	No
Liu et al. (32)	40/40	NA	NA	Rome III	NA	3	Acupressure	3–4 times daily	Zhichang(HX2); Dachang(CO7); Jiaowozhong(TF3); Jiaogan(AH6a); Sanjiao(CO17); Pizhixia(AT4); Gan (CO12); Pi(CO13); Wei(CO4); Fei (CO14); Neifenmi (CO18)	Routine treatment	Efficacy	No
Liu et al. (33)	31/31	50.16 ± 13.58/50.13 ± 11.94	E: 10/21; 12/19	Rome III	E: 5.75 ± 2.51Y; C: 5.68 ± 2.63Y	2	Acupressure	5 times daily	Zhichang(HX2); Dachang(CO7); Xiaochang(CO6); Gan(CO12); Pi (CO13); Fei(CO14)	Mosapride Citrate Dispersible	Efficacy; PAC-SYM; serum CCK; SAS; SDS; PAC-QOL	No
Wang et al. (29)	30/30	72.3 ± 4.15/71.1 ± 3.58	E: 15/15; 14/16	Rome II	NA	2	Acupressure	4–5 times daily	Jiaowozhong(TF3); Fei(CO14); Dachang(CO7); Shen(CO10); Pi(CO13); Sanjiao(CO17)	Routine treatment	Efficacy	No
Wang et al. (34)	30/30	52.31/54.18	E: 17/13; 16/14	Rome III	NA	24d	Acupressure	3 times daily	Xin(CO15); Shenmen(TF4); Jiaogan(AH6a); Dachang(CO7); Zhichang(HX2)	Routine treatment	Efficacy; Symptom score; SAS	No
Wang et al. (40)	38/38	73.8 ± 7.1/75.1 ± 7.6	E: 15/23; 10/9	Rome IV	NA	60d	Acupressure	3–5 times daily	Jiaowozhong(TF3); Dachang(CO7); Sanjiao(CO17); Pi(CO13); Fei(CO14); Jiaogan(AH6a)	Routine treatment	Efficacy; the scores of fecal traits; weekly frequency of defecation; defecation effort	No
Zhou et al. (45)	45/45	NA	NA	NA	1.31 ± 0.65 (unit not mentioned)	NA	Acupressure	3 times daily	Dachang(CO7); Xiaochang(CO6); Zhichang(HX2)	Routine treatment	Efficacy; PAC-SYM	No

E, Experimental group; C, Control group; M, Male; F, Female; M, Month; Y, Year; NDSI, Nonulcer Dyspepsia Symptom Index; NDLQI, Nonulcer Dyspepsia Life Quality Index; FDDQL, Functional Dyspepsia Quality of Life; HAMA, Hamilton Anxiety Scale; HAMD, Hamilton Depression Scale; SDS, Self-Rating Depression Scale; SAS, Self-Rating Anxiety Scale; SF-36, Short Form-36 Health Survey; BSFS, Bristol Stool Form Scale; IBS-QOL, Irritable Bowel Syndrome Quality of Life; IBS-SSS, Irritable Bowel Syndrome Symptom Severity Scale; CSBM, Complete Spontaneous Bowel Movement; PAC-SYM, Patient Assessment of Constipation Symptoms; PAC-QOL, Patient Assessment of Constipation Quality of Life; IBS-SRS, Symptom Rating Scale; VAS, Visual Analog Scale.

Outcome indicators mainly focus on treatment effect, symptom score, quality of life, psychological status score and safety, and a few studies have explored specific mechanisms. The primary outcome measure was overall response rate, as assessed by improvement in gastrointestinal symptoms. Secondary outcomes included symptom scores, which were assessed using various scales such as IBS-SSS. In addition, quality of life was measured using scales such as IBS-QOL and PAC-QOL. Some studies used the SAS and the SDS to assess changes in participants’ levels of anxiety and depression. In terms of safety, a few studies reported adverse reactions, mostly mild reactions, such as local skin redness. The remaining details are shown in Table 1.

According to the Cochrane Bias risk Assessment tool, the methodological quality items for all included studies are presented in Supplementary Figure S1. All the included studies were verified to be randomized. One randomized controlled trial detailed the method for generating the assigned sequence and was thus evaluated as low risk (30), three randomized controlled trials were categorized as high risk (29, 40, 43), while the remaining studies were classified as unknown risk. 2 RCTs were double-blinded (36, 37) and 1 RCT (39) was single-blinded, all of which were rated as low risk. Other randomized controlled trials did not provide information on whether the assigned hiding and blind procedures were carried out, resulting in an assessment of the risk of unknown bias. The resulting data were complete and therefore assessed as low risk. Since attempts to obtain the protocol or other relevant information from the first author via email, phone, or fax were unsuccessful, the risk of bias from selective outcome reports and other sources was considered low.

### 3.2 Efficacy rate

**Figure 3:**
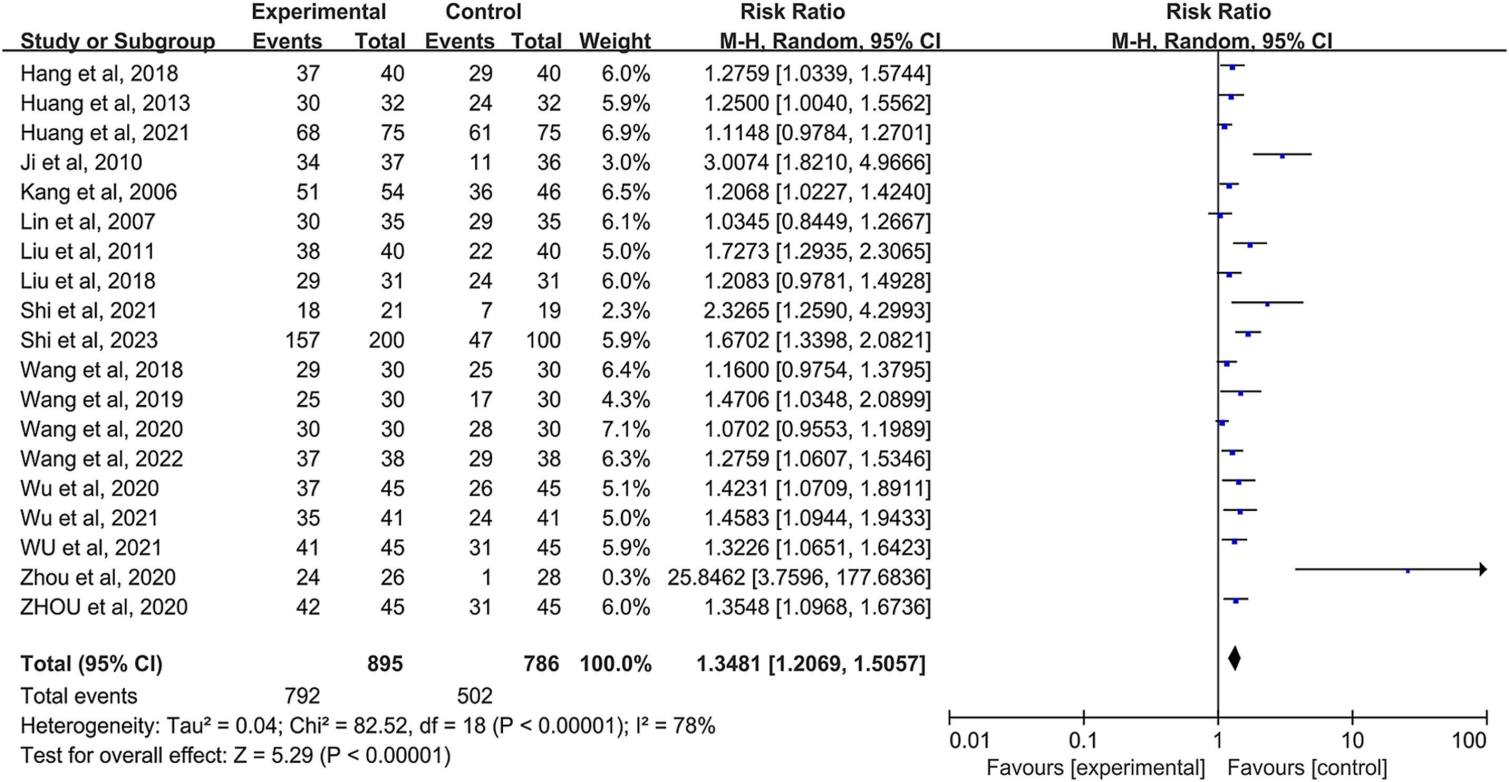
Forest plot for conventional random-effect meta-analysis.

**Figure 4:**
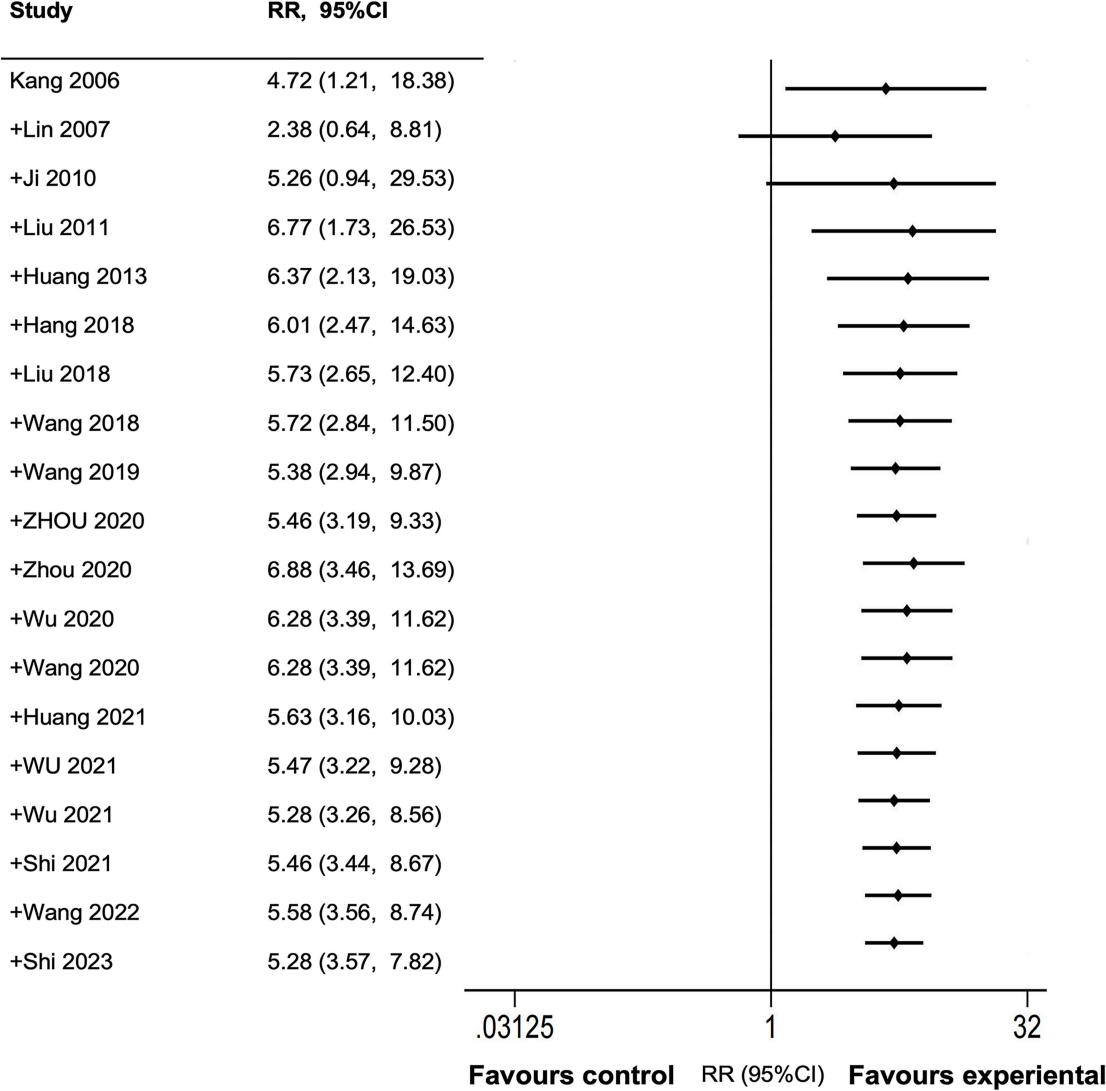
Forest plot for conventional cumulative random-effect meta-analysis.

The review included 19 studies demonstrating the effectiveness of AAT in treating FGIDs, which showed a significant therapeutic effect (*I^2^* = 78%, *p* < 0.00001) (Figure 3). Due to substantial heterogeneity, a random effects model was used. The cumulative random effects meta-analysis consistently yielded similar results (RR: 1.35; 95% CI: 1.21–1.51) even with the inclusion of newly published studies (Figure 4). We explored sources of high heterogeneity by subgroup analysis and sensitivity analysis.

The results of the subgroup analysis showed that no significant differences were found between subgroups based on types of stimulation, ear selection, number of acupoints, and intervention providers, indicating that these factors did not significantly affect clinical outcomes. In the control group, there was no heterogeneity between studies (*I^2^* = 0%), and no significant difference (*p* = 0.79) compared to conventional Western medicine treatment. However, higher inter-group heterogeneity was observed when compared with conventional care and placebo treatments (*I^2^* = 89, 70%), with statistically significant differences. Studies using fewer than five acupoints showed high inter-group heterogeneity (*I^2^* = 85%), while studies using more than five acupoints showed low inter-group heterogeneity (*I^2^* = 19%). For disease types, studies on IBS showed no inter-group heterogeneity (*I^2^ =* 0%), whereas studies on FD and FC showed high inter-group heterogeneity (*I^2^* = 86, 85%). In terms of diagnostic criteria, studies using Rome criteria showed high heterogeneity between groups (*I^2^* = 86, 85%), while studies using non-Rome criteria or other criteria showed minimal or no variability (*I^2^* = 25, 0%). These findings suggest that variation in treatment effectiveness may be related to factors such as the number of acupoints, control group measures, types of diseases, and diagnostic criteria (Table 2).

**Table 2:** Results of subgroups analyses on the effect of AAT on efficacy rate.

Subgroups	Number of studies	Number of participants (E/C)	Overall effects (RR, 95% CI)	Heterogeneity across the studies		Between-group difference (*p*-value)
				*I*^2^ (%)	*p*-value	
Stimulation type						
Acupressure	14	517/508	1.26 (1.14, 1.39)	68.0	<0.001	<0.001
Electroacupuncture	5	333/105	1.73 (1.27, 2.37)	70.0	0.01	<0.001
Disease						
Functional dyspepsia	6	381/283	1.37 (1.04, 1.81)	86.0	<0.001	0.02
Irritable bowel syndrome	4	167/159	1.26 (1.14, 1.40)	0.00	0.71	<0.001
Functional constipation	9	347/344	1.42 (1.16, 1.75)	85.0	<0.001	<0.001
Ear selection						
Unilateral application	1	26/28	1.91 (1.19, 3.07)	83.0	<0.001	0.007
Bilateral application	15	463/452	1.28 (1.15, 1.43)	71.0	<0.001	<0.001
NA	3	161/161	1.28 (1.04. 1.58)	61.0	0.08	0.02
Acupoints number						
<5	11	543/462	1.50 (1.21, 1.85)	85.0	<0.001	<0.001
≥ 5	8	287/279	1.27 (1.16, 1.39)	19.0	<0.28	<0.001
Diagnostic criteria						
Rome II	2	67/66	9.13 (3.84, 21.68)	83.0	0.02	0.05
Rome III	5	163/163	7.06 (3.13, 15.96)	72.0	0.007	0.01
Rome IV	4	150/152	7.01 (3.87, 12.69)	84.0	<0.001	0.04
Non-Rome	2	89/91	2.43 (0.99, 6.00)	25.0	0.25	0.11
NA	3	115/115	5.86 (4.19, 8.20)	0.00	0.78	<0.001
Intervention provider						
Trained therapist	4	206/206	1.29 (1.11, 1.50)	50.0	0.11	0.001
Patients	11	561/453	1.35 (1.17, 1.57)	76.0	<0.001	<0.001
Trained therapist + patients	4	128/127	1.48 (0.95, 2.32)	93.0	<0.001	0.08
Control group						
AAT versus control	6	220/219	1.49 (1.13, 1.96)	89.0	<0.001	0.005
AAT versus Placebo	6	378/278	1.73 (1.27, 2.37)	70.0	0.010	<0.001
AAT versus pharmacotherapy	7	297/289	1.16 (1.09, 1.25)	0.00	0.79	<0.001

E, Experimental group; C, Control group.

Supplementary Table S2 presents the results of leave-one-out analyses. When one study estimate was removed iteratively, the pooled effect estimates remained consistent, ranging from 1.31 (95% CI: 1.18–1.44) to 1.37 (95% CI: 1.22–1.55).

### 3.3 Symptom scores

**Figure 5:**
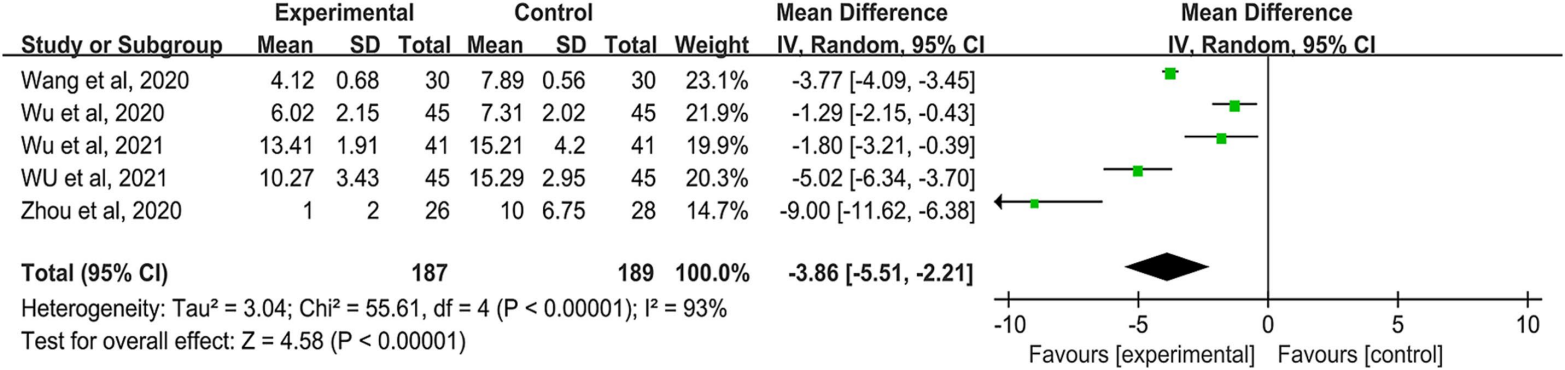
Forest plot of comparison of efficacy: symptom scores.

Five studies assessed changes in patient symptom scores after treatment, involving a total of 376 participants (34–38). The combined data showed significant heterogeneity (*I^2^* = 93%), necessitating the use of a random effects model. The review indicated that auricular stimulation significantly reduced symptom scores in FGID patients (MD: -1.94; 95% CI: −3.06 to −0.85, *p* < 0.00001) (Figure 5). Due to the observed variability, sensitivity and subgroup analyses were performed to identify potential causes. Subgroup analysis revealed varied results among studies investigating unilateral auricular acupoint compression for ear selection (*I^2^* = 86%), while other unspecified studies showed minimal or no between-group variation (*I^2^* = 0%). Significant differences persisted across other subgroups. Thus, the observed heterogeneity may be attributed to inconsistent practices related to ear selection (Supplementary Table S3). Sensitivity analysis showed minimal impact on effect estimates after sequentially excluding individual studies (Supplementary Table S4).

### 3.4 Self-Rating Anxiety Scale

**Figure 6:**
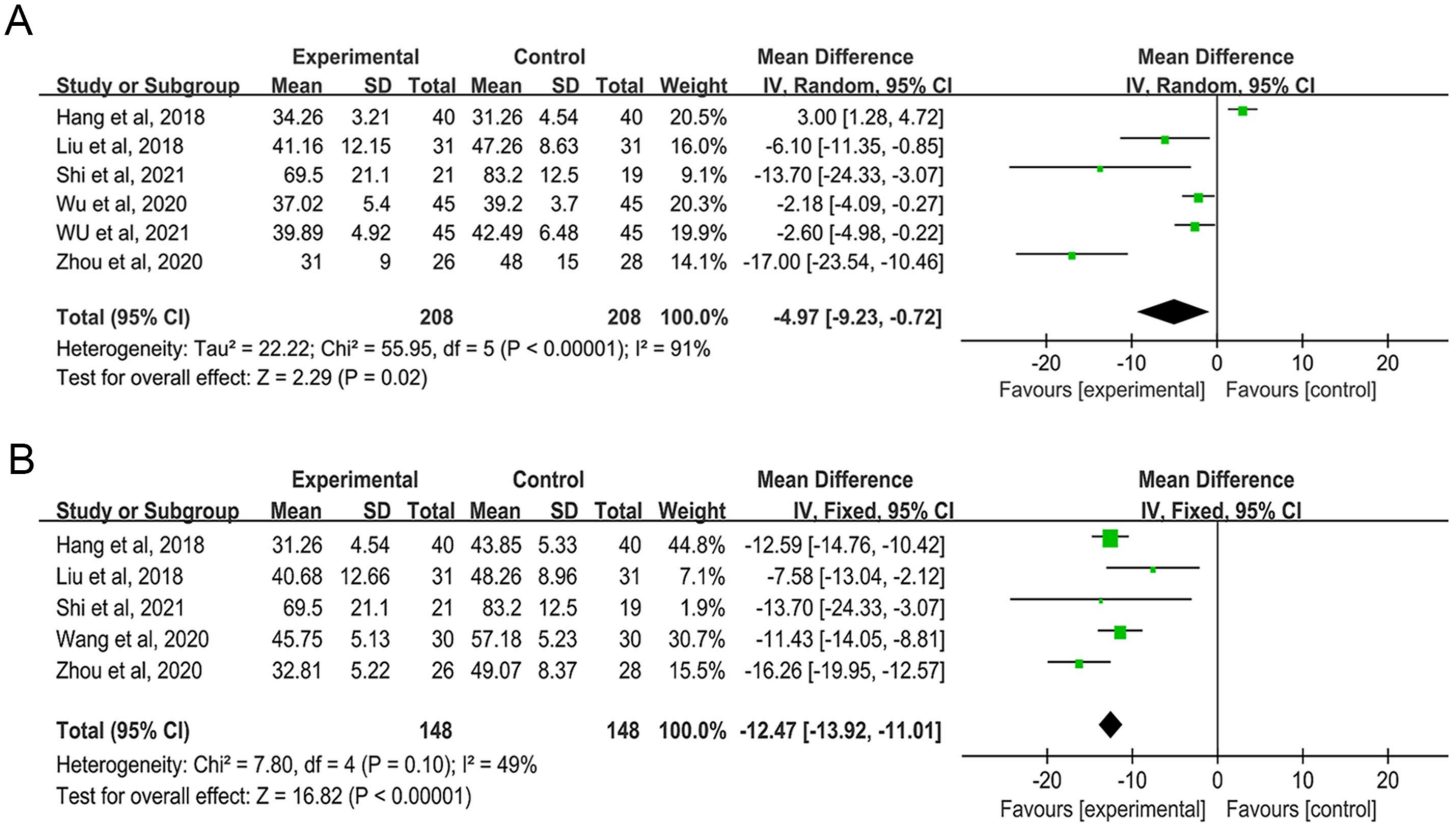
Forest plot of comparison on the effect of AAT on SAS **(A)** and SDS **(B)**.

Five studies examined changes in SAS scores among 296 participants post-treatment (33, 34, 37, 39, 43). With an *I^2^* value of 49%, indicating low heterogeneity, a fixed-effect model was used. The combined results showed that AAT significantly decreased SAS scores in FGID patients (MD: -12.47; 95% CI: −13.92 to −11.01, *p* < 0.00001) (Figure 6A).

### 3.5 Self-Rating Depression Scale

Six studies analyzed post-treatment changes in SDS scores, including 416 participants (28, 33, 35–37, 43). The heterogeneity test revealed an *I^2^* value greater than 50% (*I^2^* = 91%), leading to the use of a random effects model. The overall findings indicated that auricular point stimulation was significantly effective in reducing SDS scores (MD: -4.97; 95% CI: −9.23 to −0.72, *p* = 0.02) (Figure 6B). Subgroup analyses showed high heterogeneity, resulting in low confidence in the results (Supplementary Table S5). Sensitivity analysis confirmed minimal impact on overall outcomes after excluding individual studies (Supplementary Table S6).

### 3.6 Security analysis

Mild adverse reactions were reported in two studies. The results showed that MD = 2.98, 95% CI = (0.51, 17.26), *I^2^* = 0, *p* = 0.009. A trial (28) reported 1/37 participant in the experimental group experienced mild local skin redness due to excessive pressure on the ear point. Another trial (36) reported adverse events in 7 participants: 6/200 in the experimental group experienced transient dizziness or localized tenderness, and 1/100 in the control group reported mild headache. All of which were classified as mild and were resolved within 24 h without intervention (Supplementary Figure S2).

### 3.7 Publication bias

**Figure 7:**
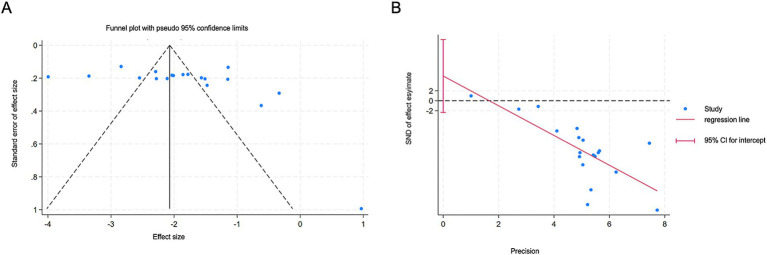
Publication bias on efficacy rate. **(A)** Funnel Plot of the efficacy rate. **(B)** Egger’s Regression Test Plot of efficacy rate.

The funnel plot for the meta-analysis of AAT for FGIDs showed symmetry. Quantitative assessment using the Egger and Begg tests revealed no significant evidence of publication bias (Egger: *β* [SE], 4.96 [3.47], *p* = 0.171) (Figure 7).

### 3.8 TSA for RCTs

**Figure 8:**
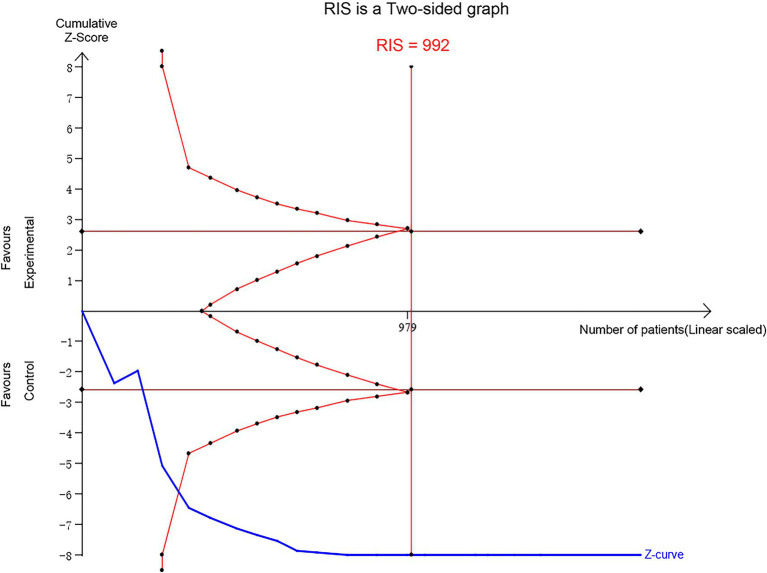
Trial sequential analysis for RCTs.

The TSA analysis revealed that the optimal sample size was 992 cases. Results showed that after the fifth study was included (28), the cumulative Z value exceeded both the traditional threshold and the TSA threshold. This finding aligns with the meta-analysis results, indicating that the sample size included in the studies has reached and surpassed the optimal value. Thus, auricular stimulation therapy for FGIDs appears to be more effective than for the control group, providing robust evidence (Figure 8).

### 3.9 Quality of evidence

The quality of evidence for all outcome measures is assessed with GRADEpro. The quality of evidence for total effective rate was low, the quality of evidence for symptom scores was low, the quality of evidence for effectiveness was rated medium, and the quality of evidence for adverse reactions was low. The quality of evidence for SAS was rated medium, and the quality of evidence for SDS was low. The most common reason for reducing the quality of evidence is high heterogeneity and high risk of bias; this is followed by inconsistency (SoF details, see Supplementary Table S7).

## 4 Discussion

To our knowledge, this systematic review is the first comprehensive analysis of AAT for FGIDs. The results suggest that AAT is effective in treating FGIDs, alleviating symptoms, and addressing anxiety and depressive states, although there is considerable statistical variability across studies. AAT can be used as a treatment option for the treatment of FGIDs.

Recently, increased attention has been given to auricular acupuncture therapy. Compared with traditional acupuncture, the use of disposable stainless steel needles will inevitably cause damage to the human body, AAT, as a non-invasive non-drug therapy, has been widely used to treat FGIDs (46). According to the “dose-effect” principle of acupuncture, higher doses of acupoint stimulation (higher frequency, multiple acupoints) are likely to produce better results (47). Consistent with this, subgroup analysis in our meta-analysis revealed significant clinical differences based on the number of acupoints used. It is worth noting that no significant difference was found in the effectiveness of interventions based on the type of AAT provider. Previous studies have utilized ear acupressure managed by nurses (48), and our results further support the effectiveness of interventions conducted by trained professionals, including nurses or collaborative teams of professionals and patients. Additionally, some studies have applied ear acupuncture to only one ear, suggesting that bilateral application might increase discomfort during treatment (49, 50). However, our study indicates that bilateral application is more common in treating FGIDs, potentially enhancing treatment intensity. Furthermore, the selection of acupoints varied across studies. Among the 19 RCTs analyzed, the most frequently used acupoints were the large intestine (*n* = 11), followed by the spleen (*n* = 8) and the liver (*n* = 7). The large intestine and liver acupoints are located in the cavum cymba conchae area, whereas the spleen acupoint is located in the cavum conchae area. These areas are commonly targeted in treatment. According to traditional Chinese medicine theory, the large intestine helps with bowel movements and diarrhea (51), while the spleen and liver regulate qi and blood, aiding in restoring organ function (52). Therefore, clinicians using AAT for FGIDs may consider bilateral application and the inclusion of these acupoints.

Different stimulation regimens may also contribute to the high heterogeneity observed in the results. Acupressure and electroacupuncture share the same stimulation mechanism-vagus nerve stimulation, but differences in nervous system activation and stimulation intensity, among others, may lead to different treatment mechanisms and effects (53). Acupressure may be more effective in promoting neurotransmitter release and mood improvement. It may be particularly appropriate for patients with mild disease who prefer noninvasive treatment. Electroacupuncture, on the other hand, may be more effective in modulating the autonomic nervous system and reducing stress, provides more consistent, controlled stimulation, and may be more appropriate for severe patients who require more intense stimulation (54). In addition, there are some differences in the efficacy of auriculotherapy for FD, IBS and FC. Our results showed that FD had the highest heterogeneity, suggesting that efficacy stability may be low, possibly due to large differences in electroacupuncture parameters (e.g., frequency, intensity) used in different studies, and differences in treatment response by different subtypes of FD (e.g., postprandial discomfort syndrome and epigastric pain syndrome) were not adequately stratified. However, IBS had the most consistent efficacy of AAT and a relatively stable improvement in anxiety and depression, which may be closely related to the central mechanism. The pooled effect size of FC was significant, but the heterogeneity was high, especially in the subgroup of “auricular number ≥ 5,” the effect was more stable, and the improvement in bowel motor function was more direct, but the effect on emotional symptoms was weaker. This may be due to the inconsistent definition of “effective” in different studies and the fact that this article includes a large number of older patients who have a weak response to treatment due to the natural decline in bowel motility.

At present, there are more and more researches on AAT in the treatment of FGIDs. Our results show that since Liu et al.’s research in 2011, with the gradual addition of new research, the 95% CI has gradually narrowed, the accuracy has improved, and the direction of evidence is consistent. Sensitivity analysis and TSA also show that the results of this meta-analysis are stable, which shows that the statistical evidence of the effectiveness of AAT in the treatment of FGIDs is sufficient. At the same time, our research results are basically consistent with other meta-analyses (55, 56). Although the differences in inclusion criteria and statistical methods may result in slightly different reported effects, they all confirm the efficacy of AAT in the treatment of FGIDs. At present, the treatment of FGIDs remains a major challenge. The existing drugs, such as prokinetic drugs and proton pump inhibitors, only treat one symptom at a time, and the combined use is easy to increase the side effects, so many problems such as long-term recurrent attacks and drug side effects have attracted more and more attention. Compared with drug therapy, AAT provides a non-invasive, safe, and minimal side-effect alternative therapy (57). Some non-drug therapies, such as cognitive behavioral therapy and hypnotherapy, have some effects, but their clinical efficacy and safety still need to be verified by large-scale clinical research (58). AAT is cost-effective, has low treatment costs, and has the potential for self-medication, reducing the need for frequent medical treatment. In conclusion, AAT is a promising alternative to medications and psychotherapy for the treatment of FGIDs, providing a safe, effective, and economical choice for clinicians and patients. Future research should further compare the efficacy, safety, and cost-effectiveness of AAT with other treatment methods to provide more comprehensive guidance for clinical practice.

However, the methodological limitations of the included studies, such as the lack of blinding and allocation concealment, high heterogeneity, and small sample sizes, resulted in most outcomes being rated as low or very low quality. These limitations restrict the generalizability of the findings to a broader population. Therefore, when interpreting the results of this study, these limitations should be taken into account, and further well-designed trials are needed to address these limitations and provide more robust and reliable evidence.

Recently, the brain-gut axis has received increasing attention. The brain-gut axis is a two-way communication system between the central nervous system and the gastrointestinal tract (59). Previous studies have shown that AAT (such as the auricular distribution area of the vagus nerve) can directly regulate autonomic nervous function by activating the vagus afferent pathway. Vagal activation can inhibit sympathetic excitability and increase parasympathetic tone, thereby reducing the systemic stress response (e.g., decreasing cortisol release) and improving visceral hypersensitivity and gastrointestinal motility disorders (60). In addition, AAT may also affect the release of neurotransmitters such as serotonin, dopamine, and norepinephrine. These neurotransmitters are involved in the regulation of mood (61). Our results suggest that AAT may not only improve symptoms in FGID patients, but also effectively alleviate anxiety and depression in FGID patients, and ear selection and intervention providers may be one of the reasons for the high heterogeneity. This study provides an evidence-based basis for non-drug intervention for the “brain-gut axis” theory. More studies combined with neuroimaging (such as fMRI) should be included in the future to explore the specific mechanism of AAT in the treatment of FGID from the perspective of the gut-brain axis.

### 4.1 Strengths and limitations

This systematic review has several notable advantages. To our knowledge, this is the first comprehensive meta-analysis that systematically summarizes and analyzes the effectiveness of AAT for FGIDs. Unlike traditional meta-analyses, the cumulative meta-analysis approach provides insights into how the pooled estimates and their precision evolve over time as new studies are added. Additionally, we performed subgroup analyses to investigate the source of heterogeneity and validated the stability of the results through sensitivity and TSA.

However, our study has several limitations. First, although most studies used uniform criteria, a few employed different efficiency standards, which might have affected the meta-analysis results. Second, our search was limited to articles in English and Chinese, which may have introduced publication bias by excluding relevant studies in other languages. Third, inadequate adjustment for key confounding variables during subgroup analyses may affect the credibility of our conclusions. Finally, all included studies were conducted in developing countries, so the generalizability of the results requires further evaluation.

## 5 Conclusion

In conclusion, AAT shows promise as a complementary therapy for FGIDs, with preliminary evidence supporting its efficacy and safety. However, the clinical implications of these findings remain provisional due to the heterogeneity and methodological limitations of existing trials. Future large-scale, rigorously designed RCTs—with standardized protocols, robust blinding, and diverse populations—are essential to confirm these results and establish evidence-based guidelines.

## Data Availability

The original contributions presented in this study are included in this article/Supplementary material, further inquiries can be directed to the corresponding authors.
