# Supplementary material for: Auricular acupoint therapy for functional gastrointestinal disorders: a systematic review and meta-analysis of randomized clinical trials
Source: Front Med (Lausanne). 2025 Mar 19;12:1513272. doi: 10.3389/fmed.2025.1513272 (PMC11962017; doi:10.3389/fmed.2025.1513272)
Supplement: Supplementary file 1 [file Data_Sheet_1.docx]

**Auricular acupoint therapy for functional gastrointestinal disorders: a systematic review and meta-analysis of randomized clinical trials**

Meng-Yuan Shen^†,1^; Ze-Jiong Li^†,1^; Shu-Han Wang^†,1^; Tian-Chen Lin^†,1^; Qin-Yi Lou^1^; Shan Liu^1^; Dan-Dan Feng^2^; Dong-Dong Yang^1^; Chen-Juan Wang^1^; Zhe-Kai Ying^1^; Rong Zhou^2^; Jian-Nong Wu^*,2^

^1^The First Affiliated Hospital of Zhejiang Chinese Medical University (Zhejiang Provincial Hospital of Chinese Medicine), Zhejiang, China.

^2^Department of Intensive Care Unit, The First Affiliated Hospital of Zhejiang Chinese Medical University (Zhejiang Provincial Hospital of Chinese Medicine), No. 54 Post Road, Shangcheng District, Hangzhou 310006, Zhejiang Province, China.

*Correspondence:

Jian-Nong Wu, MD, Chief Physician

weiyidiandian@zcmu.edu.cn

^†^These authors have contributed equally to this work

**
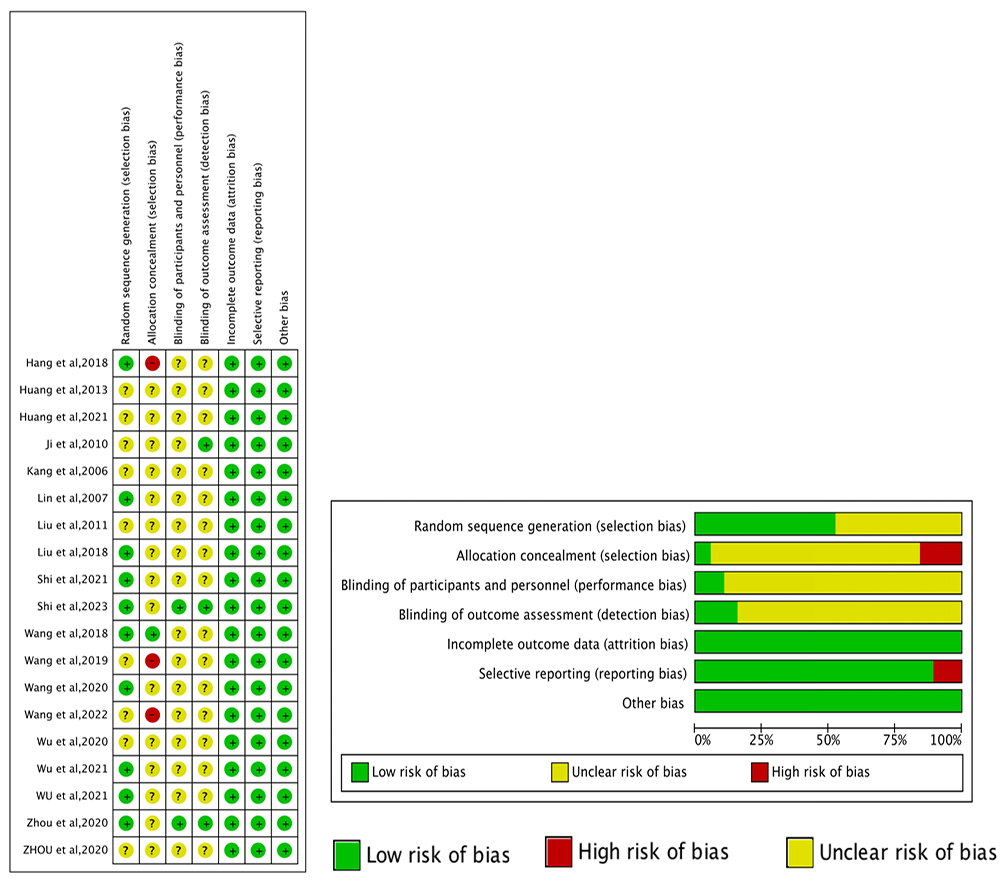
**

**Supplement Fig. 1** Summary of risk of bias

**
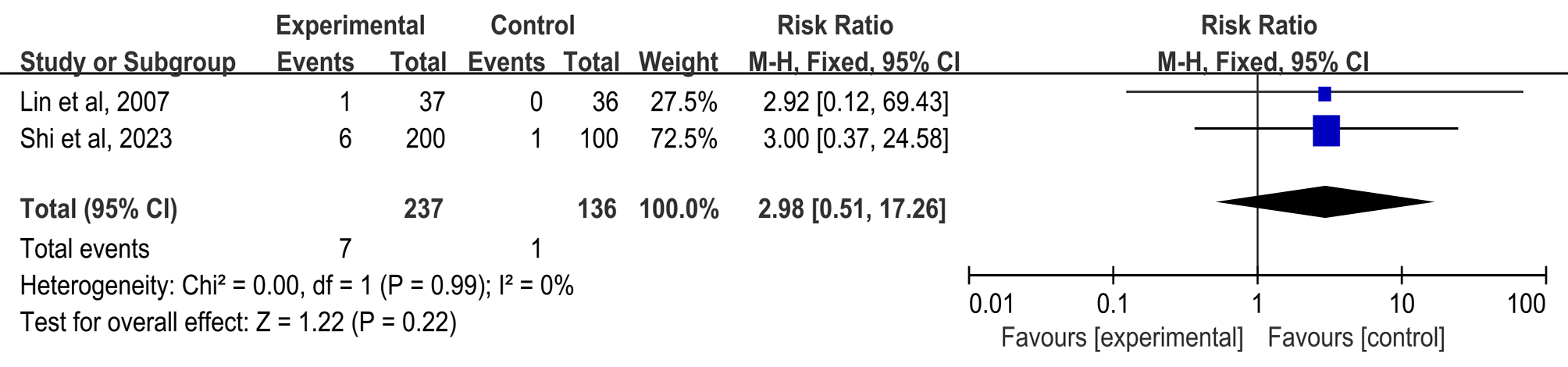
**

**Supplement Fig. 2** Forest plot of comparison of adverse events

**Table S1.** Search terms and strategy

1. **Pubmed database of systematic reviews from inception to 30/6/2024**

| **Source** | **Search Strategy** | |
| --- | --- | --- |
| **Pubmed** | **#1** | Dyspepsia [MeSH Terms] |
|  | **#2** | Indigestion [Title/Abstract] OR postprandial distress syndrome [Title/Abstract] OR epigastric pain syndrome [Title/Abstract] OR indigestion [Title/Abstract] OR functional dyspepsia [Title/Abstract] OR functional dyspepsia [Title/Abstract] |
|  | **#3** | Irritable Bowel Syndrome [MeSH Terms] |
|  | **#4** | irritable bowel syndromes [Title/Abstract] OR mucous colitis [Title/Abstract] OR mucous colitides [Title/Abstract] OR syndrome, irritable bowel [Title/Abstract] OR syndromes, irritable bowel [Title/Abstract] OR irritable colon [Title/Abstract] OR colitis, mucous [Title/Abstract] OR colitides, mucous[Title/Abstract] OR mucouscolitides [Title/Abstract] |
|  | **#5** | Constipation [MeSH Terms] |
|  | **#6** | functional constipation [Title/Abstract] OR dyschezia [Title/Abstract] OR colonic inertia [Title/Abstract] OR chronic functional constipation [Title/Abstract] OR slow transit constipation [Title/Abstract] OR chronic severe functional constipation [Title/Abstract] |
|  | **#7** | functional gastrointestinal disorders [Title/Abstract] OR functional bowel dis*[Title/Abstract] OR functional gastrointestinal dis*[Title/Abstract] |
|  | **#8** | #1 OR #2 OR #3 OR #4 OR #5 OR #6 OR #7 |
|  | **#9** | Acupuncture [Title/Abstract] OR electroacupuncture [Title/Abstract] OR bloodletting [Title/Abstract] OR laser [Title/Abstract] OR acupressure [Title/Abstract] OR acupoint [Title/Abstract] OR therapy [Title/Abstract] OR stimulate [Title/Abstract] OR press stick needle [Title/Abstract] OR auriculotherapy [Title/Abstract] OR otopoint [Title/Abstract] OR ototherapy [Title/Abstract] |
|  | **#10** | Auricular [Title/Abstract] OR ear [Title/Abstract] OR acupuncture treatments [Title/Abstract] |
|  | **#11** | #11 AND #12 |
|  | **#12** | randomized controlled trial [MeSH Terms] |
|  | **#13** | randomized controlled trial [Title/Abstract] OR random allocation [Title/Abstract] OR randomization [Title/Abstract] OR randomized controlled study OR random [Title/Abstract] |
|  | **#14** | #12 OR #13 |
|  | **#15** | #8 AND #11 AND #14 |

1. **EMBASE database of systematic reviews from inception to 30/6/2024**

| **Source** | **Search Strategy** | |
| --- | --- | --- |
| **EMBASE** | **#1** | 'dyspepsia'/exp OR 'constipation'/exp OR 'epigastric pain syndrome' OR 'postprandial distress syndrome' OR 'postprandial dyspepsiar' OR 'functional dyspepsia' OR 'dyspeptic syndrome' OR 'dyspeptic disorder' OR 'colon spasm'/exp OR 'colon spasm' OR 'colon, irritable'/exp OR 'colon, irritable' OR 'colonic diseases, functional'/exp OR 'colonic diseases, functional' OR 'colonospasm'/exp OR 'colonospasm' OR 'irritable bowel syndrome'/exp OR 'irritable bowel syndrome' OR 'irritable colon syndrome'/exp OR 'irritable colon syndrome' OR 'mucomembraneous colitis'/exp OR 'mucomembraneous colitis' OR 'mucomembranous colitis'/exp OR 'mucomembranous colitis' OR 'mucous colitis'/exp OR 'mucous colitis' OR 'spastic colitis'/exp OR 'spastic colitis' OR 'spastic colon'/exp OR 'spastic colon' OR 'unstable colon' OR 'dyschezia'/exp OR 'dyschezia' OR 'obstipation'/exp OR 'obstipation' OR 'rectal constipation'/exp OR 'rectal constipation' OR 'slow transit constipation'/exp OR 'slow transit constipation' OR 'functional constipation' OR 'functional gastrointestinal' |
|  | **#2** | ('acupuncture':ab,ti,kw OR 'acupressure':ab,ti,kw OR 'electro$acupuncture':ab,ti,kw OR 'bloodletting':ab,ti,kw OR 'laser*':ab,ti,kw OR 'therapy':ab,ti,kw OR 'acupoint*':ab,ti,kw OR 'stimulat*':ab,ti,kw OR 'press*':ab,ti,kw OR 'needl*':ab,ti,kw OR 'stick*':ab,ti,kw) AND ('ear':ab,ti,kw OR 'auricular':ab,ti,kw) OR 'otoneedle':ab,ti,kw OR 'otopoint':ab,ti,kw OR 'ototherapy':ab,ti,kw OR 'auriculo$therapy':ab,ti,kw OR 'auriculo&acupuncture':ab,ti,kw OR 'auriculo&acupressure':ab,ti,kw |
|  | **#3** | 'randomized controlled trial'/exp OR 'controlled trial, randomized' OR 'randomized controlled study' OR 'randomized controlled trial' OR 'randomized controlled study' OR 'trial, randomized controlled':ab,kw,til' OR 'random' |
|  | **#4** | [humans]/lim |
|  | **#5** | #1 AND #2 AND #3 AND #4 |

1. **Cochrane Library database of systematic reviews from inception to 30/6/2024**

| **Source** | **Search Strategy** | |
| --- | --- | --- |
| **Cochrane Library** | **#1** | MeSH descriptor: [Dyspepsia] explode all trees |
|  | **#2** | (Dyspepsias OR Indigestion OR Indigestions OR postprandial distress syndrome OR epigastric pain syndrome):ti,ab,kw |
|  | **#3** | MeSH descriptor: [Irritable Bowel Syndrome] explode all trees |
|  | **#4** | (Syndromes, Irritable Bowel OR Irritable Colon OR Syndrome, Irritable Bowel OR Mucous Colitis OR Colitis, Mucous):ti,ab,kw |
|  | **#5** | MeSH descriptor: [Constipation] explode all trees |
|  | **#6** | (Colonic Inertia OR Dyschezia):ti,ab,kw |
|  | **#7** | (functional gastrointestinal disorders): ti,ab,kw |
|  | **#8** | #1 OR #2 OR #3 OR #4 OR #5 OR #6 OR #7 |
|  | **#9** | (acupuncture OR electroacupuncture OR bloodletting OR laser OR acupressure OR acupoint OR therapy OR stimulat OR press stick):ti,ab,kw |
|  | **#10** | (auricular OR ear OR acupuncture treatments):ti,ab,kw |
|  | **#11** | #8 AND #9 AND #10 |

1. **WOS database of systematic reviews from inception to 30/6/2024**

| **Source** | **Search Strategy** | |
| --- | --- | --- |
| **WOS** | **#1** | Dyspepsia [MeSH Terms] |
|  | **#2** | indigestion OR postprandial distress syndrome OR epigastric pain syndrome OR indigestion OR functional dyspepsia |
|  | **#3** | Irritable Bowel Syndrome [MeSH Terms] |
|  | **#4** | irritable bowel syndromes OR mucous colitis OR mucous colitides OR syndrome, irritable bowel OR syndromes, irritable bowel OR irritable colon OR colitis, mucous OR colitides, mucous OR mucouscolitides |
|  | **#5** | functional dyspepsia |
|  | **#6** | Constipation [MeSH Terms] |
|  | **#7** | Functional constipation OR dyschezia OR colonic inertia OR chronic functional constipation OR slow transit constipation OR chronic severe functional constipation |
|  | **#8** | functional gastrointestinal disorders OR functional bowel dis* OR functional gastrointestinal dis* |
|  | **#9** | #1 OR #2 OR #3 OR #4 OR #5 OR #6 OR #7 OR #8 |
|  | **#10** | ear OR auricular |
|  | **#11** | acupuncture OR acupressure OR electroacupuncture OR acupoint* OR bloodletting OR laser* OR therapy OR stimulat* OR press* OR needl* OR stick* OR auriculotherapy OR otoneedle OR otopoint OR ototherapy |
|  | **#12** | #10 AND #11 |
|  | **#13** | randomized controlled trial [MeSH Terms] |
|  | **#14** | randomized controlled trial OR random allocation OR randomization OR randomized controlled study OR random |
|  | **#15** | #13 OR #14 |
|  | **#16** | "until 2024/06/30"[Date - Publication] |
|  | **#17** | #9 AND #12 AND #15 AND #16 |

1. **CNKI database of systematic reviews from inception to 30/6/2024**

| **Source** | **Search Strategy** | |
| --- | --- | --- |
| **CNKI** | **#1** | Dyspepsia OR functional dyspepsia OR irritable bowel syndrome OR constipation OR functional constipation |
|  | **#2** | acupuncture OR acupressure OR electroacupuncture OR acupoint* OR bloodletting OR laser* OR therapy OR stimulat* OR press* OR needl* OR stick* OR auriculotherapy OR otoneedle OR otopoint OR ototherapy |
|  | **#3** | ear OR auricular |
|  | **#4** | Clinical randomized controlled trial OR randomized control OR randomized OR RCT OR random |
|  | **#5** | #1 AND #2 AND #3 AND #4 |
|  | **#6** | "until 2024/06/30"[Date - Publication] |
|  | **#7** | #5 AND #6 |

1. **WanFang database of systematic reviews from inception to 30/6/2024**

| **Source** | **Search Strategy** | |
| --- | --- | --- |
| **WanFang** | **#1** | Dyspepsia OR functional dyspepsia OR irritable bowel syndrome OR constipation OR functional constipation |
|  | **#2** | acupuncture OR acupressure OR electroacupuncture OR acupoint* OR bloodletting OR laser* OR therapy OR stimulat* OR press* OR needl* OR stick* OR auriculotherapy OR otoneedle OR otopoint OR ototherapy |
|  | **#3** | ear OR auricular |
|  | **#4** | Clinical randomized controlled trial OR randomized control OR randomized OR RCT OR random |
|  | **#5** | #1 AND #2 AND #3 AND #4 |
|  | **#6** | "until 2024/06/30"[Date - Publication] |
|  | **#7** | #5 AND #6 |

1. **VIP database of systematic reviews from inception to 30/6/2024**

| **Source** | **Search Strategy** | |
| --- | --- | --- |
| **VIP** | **#1** | Dyspepsia OR functional dyspepsia OR irritable bowel syndrome OR constipation OR functional constipation |
|  | **#2** | acupuncture OR acupressure OR electroacupuncture OR acupoint* OR bloodletting OR laser* OR therapy OR stimulat* OR press* OR needl* OR stick* OR auriculotherapy OR otoneedle OR otopoint OR ototherapy |
|  | **#3** | ear OR auricular |
|  | **#4** | Clinical randomized controlled trial OR randomized control OR randomized OR RCT OR random |
|  | **#5** | #1 AND #2 AND #3 AND #4 |
|  | **#6** | "until 2024/06/30"[Date - Publication] |
|  | **#7** | #5 AND #6 |

1. **SinoMed database of systematic reviews from inception to 30/6/2024**

| **Source** | **Search Strategy** | |
| --- | --- | --- |
| **SinoMed** | **#1** | Dyspepsia OR functional dyspepsia OR irritable bowel syndrome OR constipation OR functional constipation |
|  | **#2** | acupuncture OR acupressure OR electroacupuncture OR acupoint* OR bloodletting OR laser* OR therapy OR stimulat* OR press* OR needl* OR stick* OR auriculotherapy OR otoneedle OR otopoint OR ototherapy |
|  | **#3** | ear OR auricular |
|  | **#4** | Clinical randomized controlled trial OR randomized control OR randomized OR RCT OR random |
|  | **#5** | #1 AND #2 AND #3 AND #4 |
|  | **#6** | "until 2024/06/30"[Date - Publication] |
|  | **#7** | #5 AND #6 |

**Table S2.** Result of The Sensitivity Analysis on Efficacy

| **Study omitted** | **RR (95%CI)** |
| --- | --- |
| WU 2021 | 1.35 [1.20, 1.52] |
| Hang 2018 | 1.36 [1.21, 1.53] |
| Huang 2021 | 1.37 [1.22, 1.55] |
| Huang 2013 | 1.36 [1.21, 1.53] |
| Ji 2010 | 1.31 [1.18, 1.44] |
| Kang 2006 | 1.36 [1.21, 1.54] |
| Lin 2007 | 1.37 [1.22, 1.54] |
| Liu 2011 | 1.33 [1.19, 1.48] |
| Liu 2018 | 1.36 [1.21, 1.53] |
| Wang 2018 | 1.37 [1.21, 1.54] |
| Wang 2019 | 1.34 [1.20, 1.51] |
| Wang 2020 | 1.37 [1.23, 1.52] |
| Wang 2022 | 1.36 [1.21, 1.53] |
| Wu 2020 | 1.35 [1.20, 1.51] |
| Wu 2021 | 1.34 [1.20, 1.51] |
| Shi 2021 | 1.33 [1.19, 1.48] |
| Shi 2023 | 1.32 [1.19, 1.47] |
| ZHOU 2020 | 1.35 [1.20, 1.52] |
| Zhou 2020 | 1.32 [1.20, 1.46] |
| **Combined** | **1.35 [1.21, 1.51]** |

**Table S3.** Results of subgroups analyses on the effect of AAT on symptom scores

| **Subgroups** | **Number of studies** | **Number of participants (E/C)** | **Overall effects**  **(MD, 95% CI)** | **Heterogeneity across the studies** | | **Between-group difference**  **(*P* - value)** |
| --- | --- | --- | --- | --- | --- | --- |
|  |  |  |  | ***I^2^* (%)** | ***P*-value** |  |
| **Disease** |  |  |  |  |  |  |
| Functional dyspepsia | 3 | 116/118 | -2.87 (-3.57, -2.17) | 95.0 | <0.001 | <0.001 |
| Irritable bowel syndrome | 1 | 41/41 | -1.80 (-3.21, -0.39) | NA | NA | 0.01 |
| Functional constipation | 1 | 30/30 | -3.77 (-4.09, -3.45) | NA | NA | <0.001 |
| **Ear selection** |  |  |  |  |  |  |
| Unilateral application | 2 | 71/73 | -6.84 (-10.73, -2.96) | 86.0 | <0.001 | <0.001 |
| Bilateral application | 2 | 30/30 | -3.77 (-4.09, -3.45) | NA | NA | NA |
| NA | 3 | 86/86 | -1.43 (-2.16, -0.69) | 0 | 0.55 | <0.001 |
| **Acupoints number** |  |  |  |  |  |  |
| ＜5 | 4 | 157/159 | -4.07 (-6.76, -1.37) | 93.0 | <0.001 | 0.0003 |
| ≥ 5 | 1 | 30/30 | -3.77 (-4.09, -3.45) | NA | NA | <0.001 |
| **Diagnostic criteria** |  |  |  |  |  |  |
| Rome III | 1 | 30/30 | -3.77 (-4.09, -3.45) | NA | NA | <0.001 |
| Rome IV | 4 | 157/159 | -4.07 (-6.76, -1.37) | 93.0 | <0.001 | 0.0003 |
| **Intervention provider** |  |  |  |  |  |  |
| Trained therapist | 2 | 67/69 | -3.43 (-4.67, -2.18) | 96.0 | <0.001 | <0.001 |
| Patients | 3 | 90/90 | -2.40(-3.12, -1.68) | 95.0 | <0.001 | <0.001 |
| Trained therapist + Patients | 1 | 30/30 | -3.77 (-4.09, -3.45) | NA | NA | NA |
| **Control group** |  |  |  |  |  |  |
| AAT versus Control | 1 | 30/30 | -3.77 (-4.09, -3.45) | NA | NA | <0.001 |
| AAT versus Placebo | 4 | 157/159 | -4.07 (-6.76, -1.37) | 93.0 | <0.001 | 0.0003 |

**Table S4.** Result of the sensitivity analysis on symptom scores

| **Study omitted** | **MD (95%CI)** |
| --- | --- |
| Wang 2020 | -4.07 [-6.76, -1.37] |
| Wu 2020 | -4.55 [-6.32, -2.78] |
| Wu 2021 | -4.40 [-6.33, -2.47] |
| WU 2021 | -3.60 [-5.58, -1.62] |
| Zhou 2020 | -2.97 [-4.52, -1.42] |
| **Combined** | **-3.86 [-5.51, -2.21]** |

**Table S5** Results of subgroups analyses on the effect of AAT on SDS

| **Subgroups** | **Number of studies** | **Number of participants (E/C)** | **Overall effects**  **(RR, 95% CI)** | **Heterogeneity across the studies** | | **Between-group difference**  **(*P* - value)** |
| --- | --- | --- | --- | --- | --- | --- |
|  |  |  |  | ***I^2^* (%)** | ***P*-value** |  |
| **Stimulation type** |  |  |  |  |  |  |
| Acupressure | 2 | 517/508 | 1.26 (1.14, 1.39) | 68.0 | <0.001 | <0.001 |
| Electroacupuncture | 4 | 333/105 | 1.73 (1.27, 2.37) | 70.0 | 0.01 | <0.001 |
| **Disease** |  |  |  |  |  |  |
| Functional dyspepsia | 6 | 381/283 | 1.37 (1.04, 1.81) | 86.0 | <0.001 | 0.02 |
| Irritable bowel syndrome | 4 | 167/159 | 1.26 (1.14, 1.40) | 0.00 | 0.71 | <0.001 |
| Functional constipation | 9 | 347/344 | 1.42 (1.16, 1.75) | 85.0 | <0.001 | <0.001 |
| **Ear selection** |  |  |  |  |  |  |
| Unilateral application | 1 | 26/28 | 1.91 (1.19, 3.07) | 83.0 | <0.001 | 0.007 |
| Bilateral application | 15 | 463/452 | 1.28 (1.15, 1.43) | 71.0 | <0.001 | <0.001 |
| NA | 3 | 161/161 | 1.28 (1.04, 1.58) | 61.0 | 0.08 | 0.02 |
| **Acupoints number** |  |  |  |  |  |  |
| ＜5 | 11 | 543/462 | 1.50 (1.21, 1.85) | 85.0 | <0.001 | <0.001 |
| ≥5 | 8 | 287/279 | 1.27 (1.16, 1.39) | 19.0 | <0.28 | <0.001 |
| **Diagnostic criteria** |  |  |  |  |  |  |
| Rome II | 2 | 67/66 | 9.13 (3.84, 21.68) | 83.0 | 0.02 | 0.05 |
| Rome III | 5 | 163/163 | 7.06 (3.13, 15.96) | 72.0 | 0.007 | 0.01 |
| Rome IV | 4 | 150/152 | 7.01 (3.87, 12.69) | 84.0 | <0.001 | 0.04 |
| non-Rome | 2 | 89/91 | 2.43 (0.99, 6.00) | 25.0 | 0.25 | 0.11 |
| NA | 3 | 115/115 | 5.86 (4.19, 8.20) | 0.00 | 0.78 | <0.001 |
| **Intervention provider** |  |  |  |  |  |  |
| Trained therapist | 4 | 206/206 | 1.29 (1.11, 1.50) | 50.0 | 0.11 | 0.001 |
| Patients | 11 | 561/453 | 1.35 (1.17, 1.57) | 76.0 | <0.001 | <0.001 |
| Trained therapist + Patients | 4 | 128/127 | 1.48 (0.95, 2.32) | 93.0 | <0.001 | 0.08 |
| **Control group** |  |  |  |  |  |  |
| AAT versus Control | 6 | 220/219 | 1.49 (1.13, 1.96) | 89.0 | <0.001 | 0.005 |
| AAT versus Placebo | 6 | 378/278 | 1.73 (1.27, 2.37) | 70.0 | 0.010 | <0.001 |
| AAT versus Pharmacotherapy | 7 | 297/289 | 1.16 (1.09, 1.25) | 0.00 | 0.79 | <0.001 |

**Table S6.** Result of the sensitivity analysis on SDS

| **Study omitted** | **MD (95%CI)** |
| --- | --- |
| Hang 2018 | -6.73 [-10.94, -2.53] |
| Liu 2018 | -4.78 [-9.47, -0.10] |
| Shi 2021 | -4.05 [-8.37, -0.27] |
| Wu 2020 | -6.22 [-12.24, -0.21] |
| WU 2021 | -5.95 [-11.50, -0.40] |
| Zhou 2020 | -2.59 [-6.24, -1.06] |
| **Combined** | **-4.97 [-9.23, -0.72]** |

**Table S7.** Quality assessment.

| **Quality assessment** | | | | | | | **No of patients** | | | **Effect** | **Quality** | **Importance** |
| --- | --- | --- | --- | --- | --- | --- | --- | --- | --- | --- | --- | --- |
| **No of studies** | **Design** | **Risk of bais** | **Inconsistency** | **Indirectness** | **Imprecision** | **Other**  **considerations** | **AAT** | **Control** | **Relative (95%CI)** | **Absolute** |  |  |
| **Efficacy rate** | | | | | | | | | | | | |
| 19 | Randomised  trials | Serious^a^ | Serious^b^ | Not serious | Not serious | None | 792/895 (88.5%) | 502/786 (63.9%) | RR 1.35  (1.21 to 1.51) | 224 more per 1,000  (from 134 more to 326 more) | Low^a,b^ | Critical |
| **Symptom score** | | | | | | | | | | | | |
| 5 | Randomised  trials | Serious^a^ | Serious^b^ | Not serious | Not serious | None | 187 | 189 | - | MD 3.86 lower  (5.51 lower to 2.21 lower) | Moderate^a^ | Critical |
| **SAS** | | | | | | | | | | | | |
| 5 | Randomised  trials | Serious^a^ | Not serious | Not serious | Not serious | None | 148 | 148 | - | MD 12.47 lower  (13.92 lower to 11.01 lower) | Low^a,b^ | Critical |
| **SDS** | | | | | | | | | | | | |
| 6 | Randomised  trials | Serious^a^ | Serious^b^ | Not serious | Not serious | None | 208 | 208 | - | MD 4.97 lower  (9.23 lower to 0.72 lower) | Low^a,b^ | Improtance |
| **Adverse events** | | | | | | | | | | | | |
| 2 | Randomised  trials | Serious^a^ | Not serious^b^ | Not serious | Serious^c^ | None | 7/237 (3.0%) | 1/136 (0.7%) | RR 2.98  (0.51 to 17.26) | 15 more per 1,000  (from 4 fewer to 120 more) | Low^a,b^ | Critical |

CI: confidence interval; MD: mean difference; RR: risk ratio.

Explanations:

a. Absence of description of blindness and randomization

b. Heterogeneity

c. Events < 3
